# Isolated corpora cavernosa germ cell tumor metastasis requiring complex excision and reconstruction

**DOI:** 10.1016/j.eucr.2022.102236

**Published:** 2022-09-23

**Authors:** Christine Bieber, Andrew Katims, Daniel Ranti, John P. Sfakianos, Gregory Amend

**Affiliations:** Department of Urologic Surgery, Icahn School of Medicine at Mount Sinai, 1 Gustave L. Levy Pl, New York, NY, 10029, USA

**Keywords:** Testicular cancer, Soft tissue metastasis, Inflatable penile prosthesis

## Abstract

Germ cell tumors (GCT) are rare, frequently diagnosed in men aged 20–34 years old, and have a 95% 5-year relative survival rate. Metastasis from GCT has predictable spread to retroperitoneal lymph nodes. However, in cases of scrotal violation or tumor spillage, lymphatic drainage can be altered. Beyond the retroperitoneum, the most reported extra-nodal sites of metastases include the liver, lung, brain, and bone. Here we report a case of an unusual site of metastasis to the corpora cavernosa, as well as the complex reconstruction required to preserve sexual function.

## 1 Introduction

Testicular cancer is a rare disease with a prevalence of 5.5 per 100,000 in the United States in 2020.^1^ Likely due to its high incidence in the Western world, diagnostic and therapeutic approaches for germ cell tumors (GCT) are well standardized, and overall cure rates are high even in the settings of metastatic disease. Beyond regional spread to the retroperitoneal lymph nodes, GCT commonly metastasizes to the lungs or lymph nodes of the chest and neck, hence here we report a notable case of a patient with a GCT metastatic lesion to the penile base who underwent penile mass excision and subsequent corporeal reconstruction with pericardium allograft and inflatable penile prosthesis (IPP) insertion.^2^^,^^3^

## 2 Case presentation

A 47-year-old West Indian male with a history of testicular cancer presented with a large, painless mass at the base of the penis that had been waxing and waning in size for several years.

Following testicular cancer diagnosis in 2019 in Guyana, the patient underwent a right radical orchiectomy. In August of 2020, he reported a lesion at the right penile base, which was presumed to be a recurrent GCT lesion. The patient was treated with carboplatin and etoposide in Guyana. The mass recurred in the same location, and was treated with bleomycin, etoposide, and cisplatin in 2021. However, due to severe nausea and vomiting, he was only able to tolerate two cycles.

**Fig. 1:**
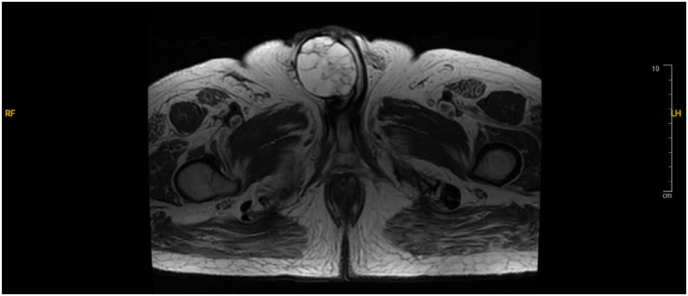
Axial T2 MRI pelvis revealed a septated mass at the base of the penis.

The patient presented later in 2021 to an outside emergency department in the United States with urinary retention and was found to have a 5.7 cm palpable mass at the base of the penis and an elevated alpha fetoprotein (AFP) of 59.4 ng/mL, wherein his care was transferred to our hospital. Biopsy-proven recurrence of non-seminomatous germ cell tumor (NSGCT) in the penis was treated with four cycles of paclitaxel, ifosfamide, and cisplatin therapy with adequate response in serum tumor markers (STMs) (AFP 871.9 → 5.8 ng/mL). One month following chemotherapy, the mass had regressed slightly in size (5.2 × 4.6 cm), but the AFP began to rise. At this time, the patient denied associated fever/chills with the mass but did endorse intermittent pain in the right groin area and difficulty voiding. The physical exam was significant for a hard, immobile, non-tender penile mass approximately 5 cm in diameter and a right inguinal scar with no palpable inguinal lymphadenopathy. The patient underwent an autologous stem cell transplant with an improvement of AFP (88.5 → 4 ng/mL). Restaging magnetic resonance imaging (MRI) demonstrated a predominantly cystic penile base lesion measuring 4.6× 4.8 cm containing multiple internal septations and no evidence of pelvic metastatic disease or lymphadenopathy (Fig. 1). The patient elected to proceed with surgical excision.

**Fig. 2:**
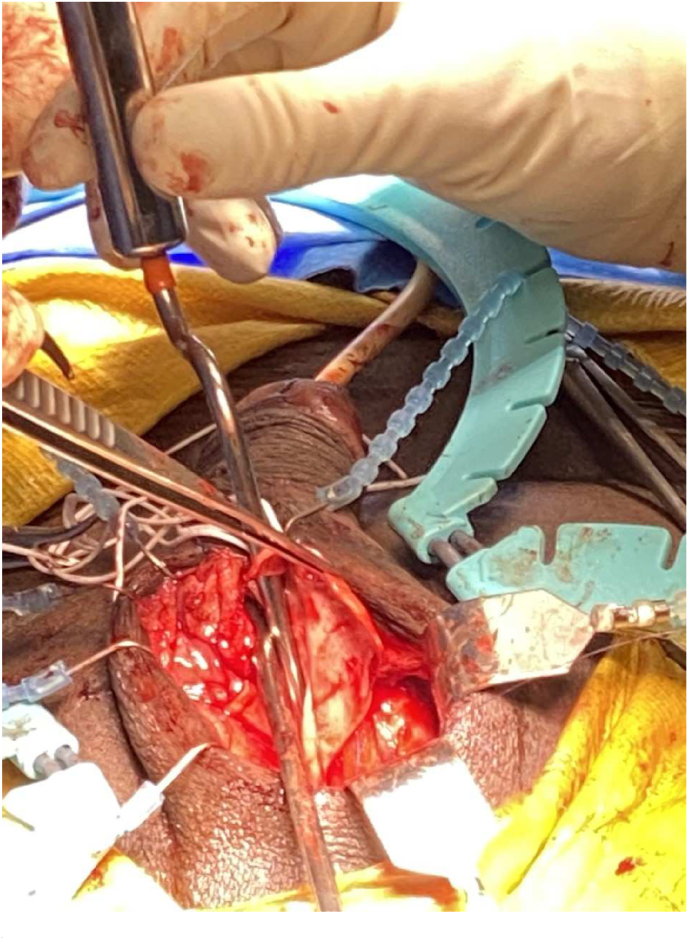
Visible defect in the right corpora following excision of the mass. The urethra is reflected with forceps. Two corporal dilators are shown entering through the proximal and distal stumps of the excised right corpora cavernosa.

**Fig. 3:**
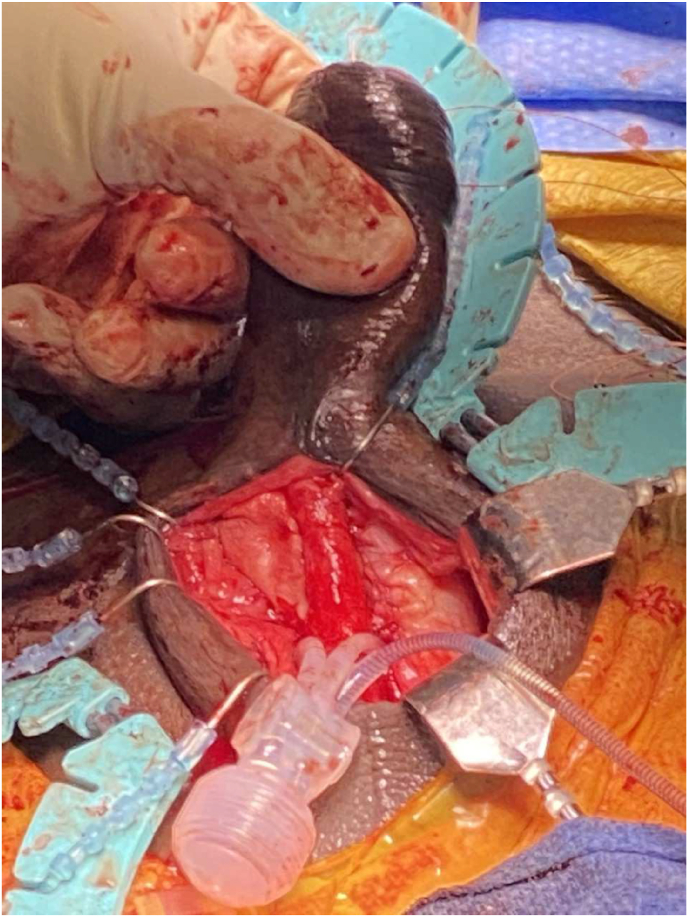
Final reconstruction of the corpora utilizing pericardium allograft with inflatable penile prosthesis.

The excision of the mass left a significant defect in the right corpora cavernosa, which was reconstructed with bovine pericardium allograft sewn to the proximal and distal cut edges (Fig. 2). The final reconstruction of the corpora is shown in Fig. 3, with pericardium allograft rolled into a tube over an inflatable penile prosthesis (IPP) on the right corpora. The left corpora cavernosa had a smaller defect which was closed primarily. The device was left in a semi-inflated state. The patient's post-operative course was uncomplicated and was discharged on post-operative day two. Pathology of the mass revealed yolk sac tumor with negative margins. The patient has since returned to Guyana and was lost to follow-up.

## 3 Discussion

This case underscores an uncommon metastatic soft tissue lesion in a patient with NSGCT that was resistant to second-line chemotherapy and stem cell transplant requiring excision. Removing the mass resulted in a significant excision of the corpora cavernosa, which required complex reconstruction with IPP placement.

There have been very few reports of GCT metastases to the soft tissues, namely of the upper arms, psoas muscle, and thighs, with variable nodal involvement and management.^4^ Bilici et al. implemented a wide excision of a soft tissue metastatic lesion to the thigh, with normalization of AFP level and no evidence of recurrence in the 10 months of follow-up postoperatively.^2^ To our knowledge, this is the first case to report a penile mass. Unusual to this presentation was a large penile lesion without regional or distal nodal involvement. Notably, the absence of detailed records of the patient's care in Guyana precludes us from staging the patient's disease and categorizing his risk; hence, we cannot determine the validity of his initial treatment plan post-orchiectomy. The patient's early relapse following first- and second-line treatment regimens warranted further management of the residual mass. Based on tumor marker kinetics, an isolated residual site of disease, and a disease state amenable to surgical resection with curative intent, the patient was an acceptable candidate for surgery.^4^

The high resectability of a penile mass was confounded by its complex invasion of the corpora and required coordinated surgical planning and operative collaboration between urologic oncology and reconstructive urology. The integration of both teams achieved adequate communication with the patient regarding oncologic outcomes and sexual function expectations, ensuring proper patient selection for graft incorporation and implantable devices given his immunocompromised status. Clearly, the oncologic benefit of a sufficient margin necessitated substitution of the corpora defect with pericardium allograft, which has been previously described in corporal reconstruction.^5^ Unfortunately the patient immediately returned to Guyana after discharge and thus the long-term functional outcome of the patient is unknown.

Consistent, interval evaluation for the early detection of recurrence and metastasis is pivotal in patients with testicular cancer, and patient compliance should be considered during shared decision-making of treatment plans. Here we highlight the unique case of a residual penile mass lesion in a patient with NSGCT and the benefits of collaboration between urologic oncologists and reconstructive urologists.

## 4 Conclusion

Interval evaluation for evidence of recurrence and metastasis in patients with testicular cancer should include the soft tissue and may require a multidisciplinary treatment team.

## Funding

This research did not receive any specific grant from funding agencies in the public, commercial, or not-for-profit sectors.

## Declaration of competing interest

The authors have no conflicts of interest to declare.
